# The Practical Medical Series of Year Books

**Published:** 1902-04

**Authors:** 


THE PRACTICAL MEDICAL SERIES OF YEAR BOOKS. Ten volumes. Issued monthly, under the general editorial charge of GUSTAVUS P. HEAD, M. D., Professor of Laryngology and Rhinology in the Chicago Post-Graduate Medical School. Vol. I. General Medicine. Edited by Frank Billings, M. S., M. D., Head of the Medical Department and Dean of the Faculty of Rush Medical College, Chicago. With the collaboration of S. C. Slanlen, M. D. October, 1901. Pp. 275. Chicago: The Year Book Publishers, 40 Dearborn Street. [Cloth, $1.50.]

The present volume is one of a series of books issued at monthly intervals, and covering the entire field of medicine and surgery, under the general editorial charge of Gustavus P. Head, M.D., professor of laryngology and rhinology in the Chicago Post-Graduate Medical School. This volume devoted to general medicine is edited by Frank Billings, M.S., M.D., dean of the faculty of Rush Medical College, Chicago. The scope of this volume is a review of the year's work in general medicine, including the results and conclusions of the best essays published in medical literature throughout the world. The diseases of the respiratory organs are granted first notice and the literature dealing with pulmonary tuberculosis is fully considered. The papers read at the British Congress on Tuberculosis which met in London during July, 1901, are abstracted and Koch's latest doctrine criticised in some of its details. Short chapters are then devoted to pneumonia, the diseases of the bronchi and the pleura. Diseases of the circulatory organs follow with a rather extended reference to diseases of the heart. The bubonic plague and variola are fully considered under the general infectious diseases, while rheumatic fever, joint affections, diabetes, osteomalacia and uric acid diathesis are taken up under the head of constitutional diseases. The blood diseases, kidney and bladder affections, obesity, cancer and headache, are reviewed under the head of miscellaneous diseases.

It is the intention to publish a second volume dealing with general medicine in May containing the literature of the year upon those diseases which are most prevalent in the summer and autumn, namely, gastro-intestinal diseases, diseases of the liver and pancreas, typhoid fever, yellow fever, dysentery and parasitic diseases. The cost of the entire set, $7.50, is within the reach of all and will appeal to many unable to secure the more expensive volumes. W.C.K.

